# A rare case of mixed gonadal dysgenesis in adulthood: diagnostic delay and unique outcomes

**DOI:** 10.1093/jscr/rjad272

**Published:** 2023-05-22

**Authors:** Ploywarong Rueangket, Supreechaya Phansenee, Nutthaporn Laoharojvongsa, Worawat Boonyachan

**Affiliations:** Department of Obstetrics and Gynecology, Phramongkutklao Hospital, Bangkok, Thailand; Department of Obstetrics and Gynecology, Phramongkutklao Hospital, Bangkok, Thailand; Department of Pathology, Army Institute Pathology, Bangkok, Thailand; Department of Obstetrics and Gynecology, Phramongkutklao Hospital, Bangkok, Thailand

## Abstract

Mixed gonadal dysgenesis (MGD) is a rare sex development disorder, diagnosed by mosaic karyotype of 45,X/46,XY (classical form) with the presence of Müllerian structures, unilateral testis and contralateral streak gonad. MGD expresses diverse phenotypes, from female phenotype with virilization or turner stigmata, to ambiguous genitalia or male phenotype. Early diagnosis is crucial for effective correction of height, sexual development and cancer prevention. The study reports on a case of a 25-year-old patient, reared as female, presenting a large abdominal mass later confirmed as a mixed germ cell tumor. Associated findings were primary amenorrhea, ambiguous genitalia, short statue, gender dysphoria and hyperlipidemia. The study is the first to report on hyperlipidemia in MGD.

## INTRODUCTION

Mixed gonadal dysgenesis (MGD) is characterized by mixed forms of defective gonadogenesis, resulting in unilateral testis, contralateral streak gonad and persistent Müllerian structures. Multiple cell lines can sporadically emerge via chromosome missegregation during early embryonic mitosis (e.g. anaphase lag or interchromosomal rearrangement). Though diagnosis confirms 45,X/46,XY (classical form) mosaic karyotype [1], precise determination requires expertise due to phenotype varieties. Although ambiguous genitalia are usually observed at birth or before puberty, our case reports a MGD diagnosed in adulthood, with large abdominal mass suspected to be gynecological cancer.

## CASE REPORT

A 25-year-old female with large abdominal mass and distension symptom was referred to our hospital’s gynecology clinic. Detailed history-taking revealed primary amenorrhea. She had an unremarkable antenatal and family history. According to her parents, her external genitalia (clitoris-like) were slightly large but unnoticed by physician at birth. She was diagnosed as phenotypic female and reared as a girl without further evaluation. There were no childhood illnesses, and her developmental milestones were average. In psychosexual development, at 8 years old, her gender identity and role were developed as a boy. At 12, she identified as a homosexual and was sexually active with a female partner. Her lack of concern for her absence of menstruation delayed her seeking medical consultation.

In the physical examination, her height was 147 cm with a mid-parenteral height of 157.5 cm. She weighed 43.6 kg with a body mass index of 20.2 kg/m^2^. Her arm span was 149 cm, and had no acne, nor deepening voice. Evaluation with Modified Ferriman–Gallwey score revealed mild hirsutism, with the score of 3 only at the upper lip area. She had no obvious Turner stigmata, nor dysmorphic features.

**Figure 1:**
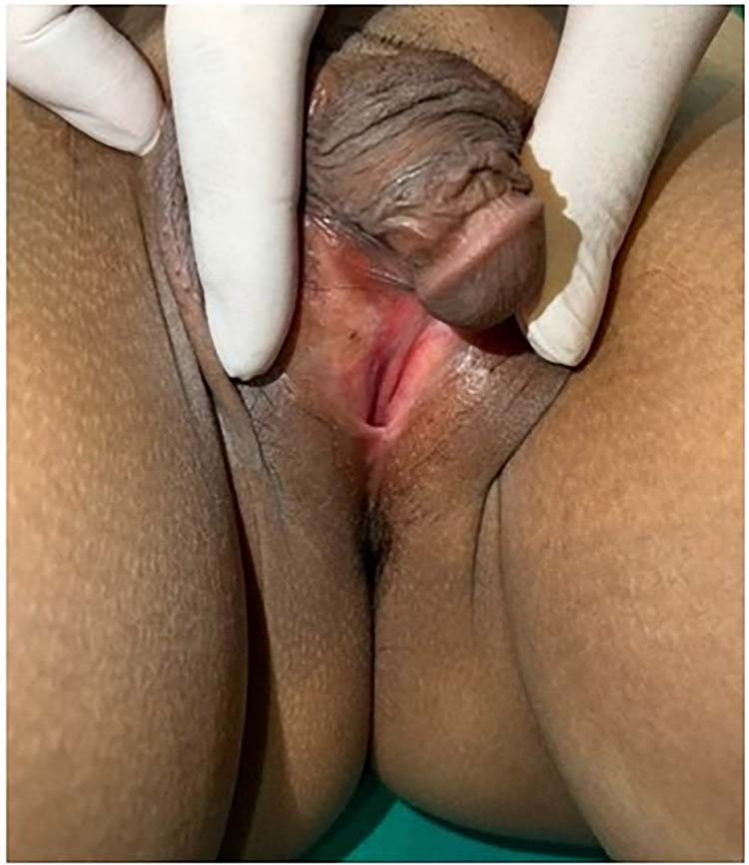
External genitalia with abnormalities: phallus 4 cm. in length, hypospadias and narrowing of vaginal orifice.

Breast development was Tanner stage I, while pubic hair Tanner stage IV. Abdominal exam found a 20-week-sized pelvic mass with firm consistency without tenderness. During the pelvic examination (Fig. 1), a phallus-like structure measuring 4 cm in length and 1.5 cm in width was found, with no labioscrotal fusion but with narrowing of the vaginal orifice and hypospadias. A possible uterus was also palpated during rectovaginal examination. Other exams were unremarkable.

Laboratory findings revealed the hypergonadotropic hypogonadism state as following: FSH level 64.9 mIU/ml and an E2 level < 20 ng/dL. Thyroid-stimulating hormone and prolactin level were normal. The androgen panel was abnormally high for female range (2.76 ng/ml), while considered within the male range but with an abnormally low baseline (≤12 nmol/L [3.50 ng/ml]). LDL was 220 mg/dl, cholesterol 299.3 mg/dl, HDL 66.2 mg/dl and TG 94.4 mg/dl.

Abnormally high level of three serum tumor markers was suspected of germ cell tumor: LDH 1740 U/L, AFP 5552 ng/ml, hCG 256 mIU/ml.

Abdominal imaging revealed a large lobulated mass measuring 15.3 × 13.6 × 8.6 cm (suspected left-ovary in origin due to venous drainage to the left ovarian vein), a small uterus and the absence of right ovary with suspicious intra-abdominal testis (2.5 cm in diameter), at the right-side of the pelvis. No sign of invasion to adjacent organs or metastasis was reported. A transrectal ultrasound for uterine evaluation suggested a probable prepubertal-sized uterus (4 cm in length).

In karyotype, 50 metaphases of peripheral blood lymphocytes were assessed with G-bands by trypsin, using Giemsa chromosome analysis, which resulted in 45,X [5]/ 46,XY[45].

**Figure 2:**
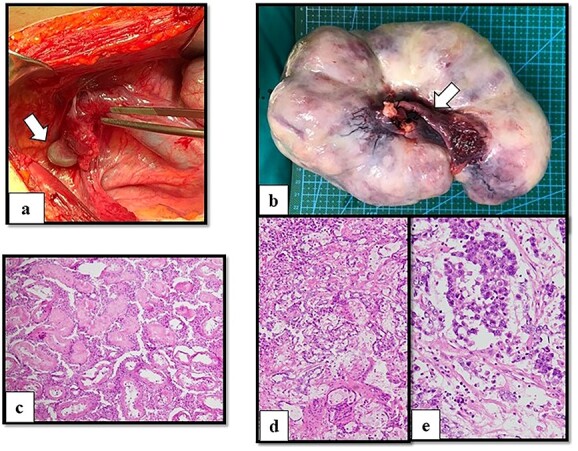
Operative and histological findings. (**a**) Macroscopic findings of right gonads (arrow) on right lateral pelvic side wall and (**b**) left gonadal tumor. A Müllerian structure-like, fallopian tube was evident (arrow). Histological examination of (**c**) atrophic testis and mixed germ cell tumor arising from streak gonad with (**d**) yolk sac tumor (**e**) and dysgerminoma. Although seminiferous tubules were identified in the testis, fibrous tissue was detected in the streak gonad.

**Figure 3:**
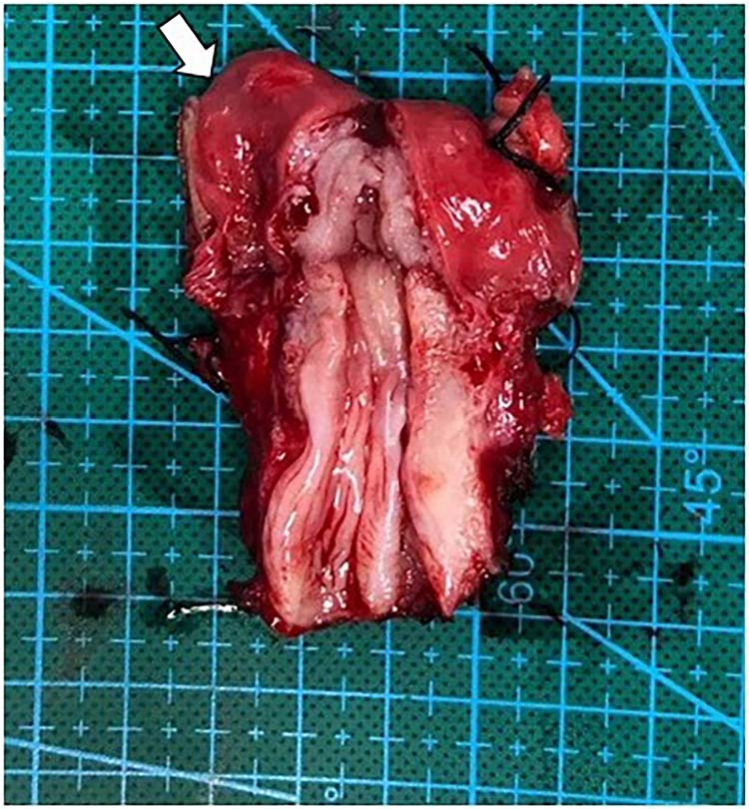
Operative finding of dissected uterus with prepubertal-stage appearance (proportion of cervix equally to uterus) and right fallopian tube (arrow).

After multidisciplinary professionals’ evaluation, MGD with gonadal malignancy was suspected. Gynecologists and urologists prepared and operated an exploratory laparotomy for left gonadal tumor resection, a right gonadectomy and a hysterectomy. The surgical findings revealed a left gonadal tumor measuring 17.5 cm in diameter, with a tan-white smoothly lobulated surface and a mix of tan-white firm contents and fleshy necrotic tissue (Fig. 2). Surgical staging was in cancer stage IC3. The pathology report revealed a right atrophic testis and a left gonadal cancer consisting of a mixed germ cell tumor (80% predominately dysgerminoma, 20% yolk sac tumor) with capsule invasion. Pathological findings of uterus and fallopian tube were normal (Fig. 3).

Postoperative systemic adjuvant chemotherapy, comprising bleomycin, etoposide and cisplatin (BEP regimen), was initiated promptly. After the fourth cycle of chemotherapy, her tumor markers were normalized, with no evidence of recurrence.

With psychiatric and endocrinological support, the sex reassignment was concluded as male. The gender transformation was planned with post-treatment hormonal therapy.

After completing the cancer treatment, gender reassignment and gender affirming hormone therapy were initiated following multidisciplinary team counseling and with the patient’s approval. Intramuscular testosterone replacement therapy was prescribed monthly. Lastly, although screening for other Turner syndrome’s comorbidities was normal, continuing surveillance was arranged.

## DISCUSSION

MGD is a complex sex chromosome abnormality, which genetically influences internal sex organ development. The phenotypic expression of anatomical and functional disturbances does not merely involve reproductive tract but multisystem. MGD is expected to cause gender dysphoria, gonadal tumors, developmental growth issues and currently is suspected to contribute to hyperlipidemia with atherosclerotic cardiovascular disease (ASCVD) risk. Due to adulthood presentation in our case, late outcomes were observed conclusively in four main aspects.

1. **Gonadal malignancy** is reported to be highest in MGD among other disorder of sex differentiation (DSD), to which the presence of Y-chromosome is significantly related. Moreover, ambiguous phenotype in our case was found to be associated with higher carcinoma risk compared with mildly undervirilized male phenotype. This might be because tumors tend to be most pronounced in immature and/or poorly differentiated gonad. Thus, the degree of testicularization of the gonad is reflected by the expressed phenotype [2]. Meanwhile, incidence of tumor increases significantly with advancing age, by 3–4% at age 10, 10–20% at age 15 and as high as 46% at age of 40 [3]. Though benign gonadoblastoma is more common, malignant germ cells arising from streak gonad occurred in our case. Thus, preventive gonadectomy must be considered before neoplastic transformation arises from streak gonad.
2. **Gender dysphoria**: In individuals with 46 XY cell lines, the partial function of the Y-chromosome may contribute to the development of unilateral testis. Dysgenesis of Sertoli cells causes retention of Mullerian ducts and impairment of testicular androgenic function, resulting in incomplete masculinization of external genitalia and secondary sex characteristics. As MGD is usually diagnosed in childhood, a multidisciplinary team should manage sex assignment. Incongruity between assigned sex and experienced gender led to gender dysphoria in our case. Although gender dissatisfaction occurs in general population, it is more common in DSD patients. Predicting gender identity from karyotype, androgen exposure, genital virilization or assigned gender remains a challenge. The association between the rearrangement of Y-chromosome and gender is currently inconclusive [4, 5]. In our case, testosterone therapy is administered for secondary sex characteristics and bone-loss prevention, alongside psychological support. However, due to the presence of both internal sex organs and ambiguous genitalia, the decision regarding sex assignment should be made carefully after thorough discussion involving experts, the patient and the family to prevent gender dysphoria.
3. **Short stature** is currently known to be related to 45,X cell by haploinsufficiency of the SHOX gene (one of the major growth regulating gene) [6]. Despite the less predominant 45,X cell population (10%) in this case, short stature still occurred, highlighting the dissociation between percentage of cell lines and growth development. Given the patient’s dissatisfaction with the short stature as a male, the MGD patient with growth deficiency would benefit from early diagnosis and initiation of treatment with growth hormone [7].
4. **Hyperlipidemia**: Although multiple mechanisms underlie hyperlipidemia, sex chromosome and gonadal hormone may profoundly influence the development path leading to a lifelong consequence by gene expression. XO compared with XX women show increased body fat, whereas in 45,X Turner syndrome, dyslipidemia is prevalent since childhood [8]. Thus, the absence of X inactivation escape genes was believed to exhibit traits of dyslipidemia. Furthermore, due to the 46,XY cell lines, a hypogonadal state in MGD caused by partial function testis is inevitable, leading to low testosterone level as reported in our case. To date, scientific data show that total and LDL cholesterol inversely correlate with testosterone levels, and low testosterone concentrations correlate with adverse cardiometabolic effects such as inflammation, insulin resistance, dyslipidemia and atherosclerosis [9, 10]. Given the patient’s high LDL and cholesterol, the ASCVD Risk Estimator Plus reports a high lifetime risk (39%), despite the patient’s average-fat diet routine without known familial hyperlipidemia. To our knowledge, metabolic defect or hyperlipidemia has never been reported in previous MGD literature. The impact of sex chromosome and gonadal hormone in DSD is inconclusive. ASCVD could be a leading cause of morbidity and mortality in MGD patients worldwide. Further study is needed for a more accurate interpretation.

## CONCLUSION

The unique outcomes in adult MGD with delayed diagnosis include not only gender dysphoria but also malignant gonadal tumor, growth failure and hyperlipidemia. A possible impact of sex chromosome in MGD on lipid metabolism was found. Due to the long-term ASCVD risk and its consequence on the overall health, intensive surveillance for dyslipidemia is recommended to enable appropriate preventive care for MGD patients.

## Data Availability

Data sharing is not applicable to this article as no new data were created or analysed in this study.
